# Regenerated lignocellulosic material from pea, bean, and artichoke waste as feedstock for bioplastic production

**DOI:** 10.1016/j.jare.2026.01.056

**Published:** 2026-01-25

**Authors:** Cyrine Amara, Safa Baraketi, Yosr Barchouchi, Yassine M’Rabet, Wiem Ayadi, Mabrouk Horchani, Khaoula Khwaldia

**Affiliations:** a Laboratory of Natural Substances (LSN), National Institute of Research and PhysicoChemical Analysis (INRAP), Sidi Thabet 2020, Tunisia; b National Agronomic Institute of Tunisia, University of Carthage, 43 Charles Nicolle Avenue, Tunis 1082 Tunisia; c Laboratory of Heterocyclic Chemistry, Natural Products and Reactivity (LR11Es39), Medicinal Chemistry and Natural Products, Faculty of Sciences of Monastir, University of Monastir, Monastir, Avenue of Environment, Monastir 5000, Tunisia

**Keywords:** Vegetable waste, Deep eutectic solvent (DES), Lignocellulosic biomass, Bioplastics, Molecular docking, Circular economy

## Abstract

**Introduction:**

Vegetable processing generates large quantities of lignocellulosic waste that are often underutilized. Developing bioactive, biodegradable packaging materials from such residues aligns with circular bioeconomy goals. This study explores oxalic acid-choline chloride deep eutectic solvent (DES) fractionation as a green approach to valorize artichoke bracts, pea pods, and bean pods into high-performance lignocellulosic bioplastics.

**Objectives:**

The aim was to regenerate lignocellulosic matrices in situ and fabricate bioplastics with tailored structure–property relationships, while providing mechanistic insights into the molecular interactions governing their performance.

**Methods:**

Raw wastes were subjected to DES fractionation, which selectively reorganized cellulose, depolymerized hemicellulose, and regenerated lignin. Fourier-transform infrared (FTIR) spectroscopy and scanning electron microscopy (SEM) were used to confirm structural changes. Mechanical, barrier, thermal, antioxidant, antimicrobial, and biodegradation properties were evaluated. Molecular docking simulations were performed to investigate lignin-cellulose interactions.

**Results:**

DES treatment produced dense fibrillar networks in artichoke-derived bioplastics and more porous architectures in pea and bean pod bioplastics. Molecular docking indicated favorable lignin-cellulose interactions (binding energies − 4.9 to − 5.2 kcal/mol) stabilized by oxalic acid-mediated hydrogen bonds, supporting enhanced matrix cohesion. Artichoke bract bioplastics exhibited the highest tensile strength (7.02±0.68 MPa) and Young’s modulus (428.11±44.47 MPa), whereas bean pod bioplastics achieved the highest elongation at break (8.35±1.30 %). Pea pod bioplastics offered superior water vapor barrier properties. Thermal stability was enhanced (T_d_ = 244–266 °C), UV–Vis light transmittance was below 2 %, and the films retained antioxidant and antimicrobial activity. Soil burial tests showed 56–69 % mass loss within 70 days, indicating rapid biodegradability.

**Conclusion:**

This work demonstrates that DES-mediated regeneration of lignocellulosic material is an effective strategy to produce multifunctional, bioactive, and environmentally degradable bioplastics. By correlating molecular-level lignin-cellulose interactions with macroscopic mechanical, barrier, and bioactive properties, the study provides mechanistic insight and practical guidance for the rational design of sustainable packaging materials within a circular bioeconomy framework.

## Highlights

• Vegetable wastes were valorized into lignocellulosic bioplastics using DES treatment.
• Molecular docking confirmed lignin–cellulose interactions stabilizing bioplastic matrix.
• DES-mediated regeneration enabled tunable mechanical behavior, combining strength and flexibility.
• All bioplastics exhibited excellent UV shielding (>98 %).
• Bioplastics retained antioxidant activity, showed antimicrobial effects, and biodegraded fast.

## Graphical abstract

**Figure:**
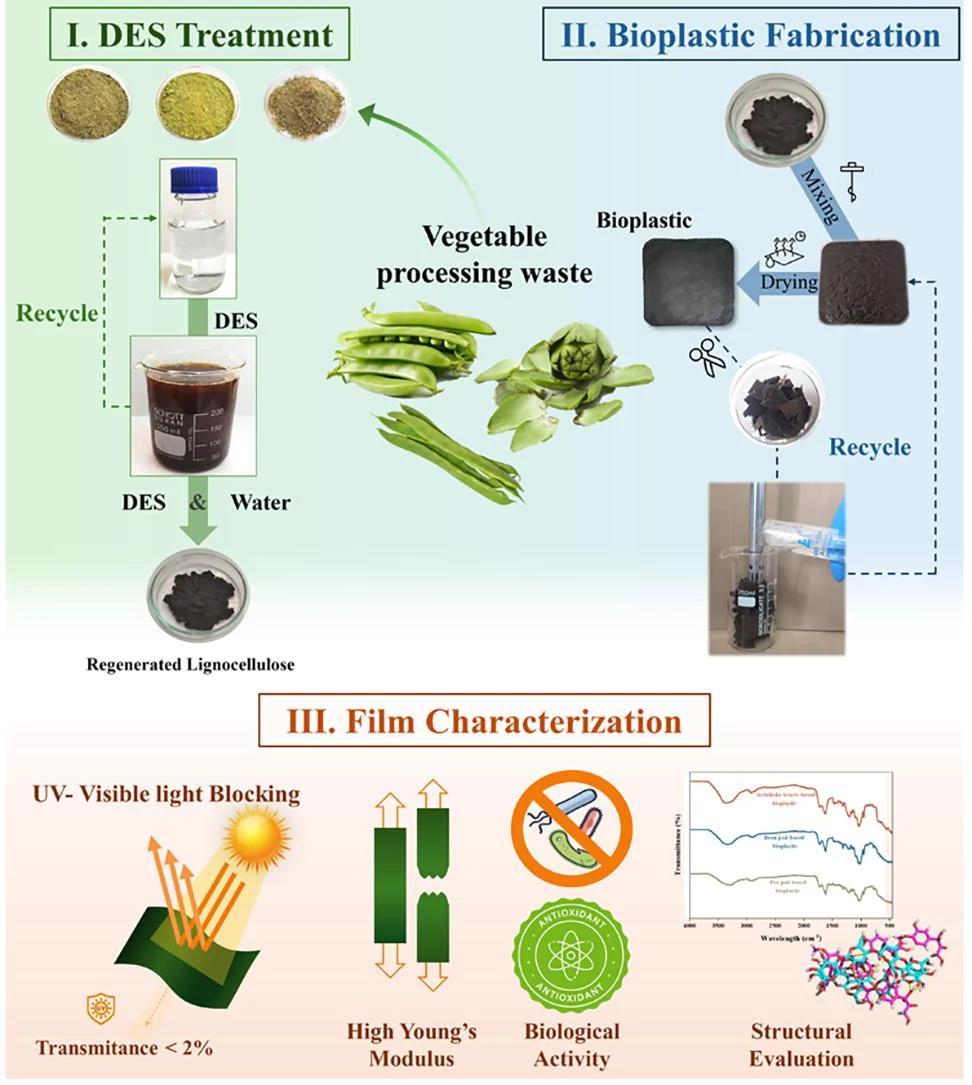

## Introduction

Synthetic plastics have become integral to modern society, owing to their adaptable properties, long lifespan, and affordability. However, the vast majority are derived from fossil-based raw materials, contributing not only to the depletion of non-renewable resources but also to significant greenhouse gas emissions [1]. Since the mid-20^th^ century, global plastic production has surged from a few million tonnes to over 460 million tonnes in 2024, marking a 230-fold increase since 1950, with approximately half of this volume destined for single-use applications [2]. These petroleum-based polymers are highly resistant to degradation and can persist in the environment for centuries. As a result, approximately 5 billion tons of plastic waste have already accumulated worldwide, with projections estimating 11 billion metric tons by 2025 [1,2]. Their persistence promotes the formation of micro- and nanoplastics, emerging contaminants that infiltrate terrestrial and aquatic ecosystems, enter food chains, and pose risks to plant, animal, and human health [3]. Moreover, the incineration of plastic waste leads to the emission of hazardous and toxic gases, further exacerbating environmental degradation and harming biodiversity [4].

To address the escalating plastic pollution crisis, there is an urgent need to replace conventional petroleum-based plastics with environmentally friendly alternatives. Bioplastics, particularly those derived from renewable resources, offer a promising solution by reducing dependence on fossil fuels and lowering greenhouse gas emissions [5]. Among these, agri-food waste and by-products, particularly from fruits and vegetables, are attractive feedstocks due to their richness in organic and bioactive compounds. Despite their large quantities, these residues remain largely underutilized. Their valorization into sustainable packaging materials not only contributes to waste reduction but also supports the transition to a circular economy while creating new economic opportunities [2,5]. Global food waste, estimated at $400 billion annually, entails major losses in water, land, and energy resources [6]. Recognizing its environmental and economic consequences, governments, including the European Union, have developed strategies to improve food system efficiency and mitigate food loss and waste [7]. Such efforts are also gaining momentum in North African countries such as Tunisia, where the agri-food industry generates significant quantities of underutilized organic waste. National initiatives have been introduced to improve waste valorization and strengthen food system resilience [8].

The development of bioplastics from lignocellulosic biomass has gained increasing attention as a sustainable alternative to petrochemical polymers [5]. However, conventional fabrication methods often rely on complex and energy-intensive processes such as delignification [9], chemical crosslinking [10], or fiber modification [11]. These approaches typically involve toxic reagents, multiple purification steps, and costly additives, which limit their environmental and economic viability [2]. Moreover, improving the mechanical strength and water resistance of bio-based films remains a significant challenge, primarily due to the hydrophilic nature and poor interfacial bonding of cellulose and hemicellulose [11]. Although the incorporation of micro- or nanoscale lignocellulosic components has shown potential to enhance certain properties, the use of synthetic adhesives and harsh processing conditions undermines the sustainability of such materials [12]. Therefore, there is a pressing need to develop greener fabrication strategies that enhance material performance without compromising environmental goals.

In this context, our study proposes a simple and eco-friendly method based on deep eutectic solvent (DES)-assisted in situ lignin regeneration, which bypasses lignin-cellulose separation, a process that is typically labor-intensive, energy-consuming, and waste-generating. By preserving lignin within the biomass matrix, this strategy exploits its natural role as a binding agent, enabling the formation of dense, cohesive bioplastics with enhanced mechanical properties, improved water and thermal stability, and reduced production costs [13]. Beyond this general concept, the present work advances current knowledge by explicitly linking the compositional variability of different agro-wastes to structure-property relationships in regenerated lignocellulosic bioplastics. While in situ lignin regeneration has recently emerged as a promising approach for fabricating high-performance lignocellulosic bioplastics, reported studies have focused mainly on relatively homogeneous lignocellulosic feedstocks such as wood [13], corn stover [12], bamboo leaf powder [14]. Consequently, the influence of feedstock-specific lignocellulosic composition (cellulose, hemicellulose, and lignin balance) on matrix reorganization, mechanical behavior, and functional performance remains insufficiently understood. Moreover, mechanistic insights at the molecular scale describing how regenerated lignin interacts with cellulose following DES treatment are rarely addressed [15,16]. To address this gap, a green and cost-effective in situ lignin regeneration strategy assisted by DESs was applied to three types of vegetable processing waste, pea pods, bean pods, and artichoke bracts, which are abundantly available yet unexplored as sources for bioplastic production. These residues differ markedly in their native lignocellulosic composition, offering a unique platform to elucidate how compositional heterogeneity governs matrix restructuring and end-use properties [15]. DESs, recognized for their biodegradability, low toxicity, and recyclability, facilitate efficient disruption of lignin-cellulose interactions and promote regeneration of a self-binding cellulose-lignin network without requiring complete separation or purification [[12], [13], [14]].

In addition to conventional structural and performance characterization, this study introduces molecular docking as a complementary tool to rationalize lignin-cellulose interactions at the molecular level following DES treatment, providing mechanistic insight that is largely absent from prior DES-based bioplastic studies. To the best of our knowledge, this is the first work to combine vegetable processing wastes with in situ lignin regeneration and molecular-level analysis to produce multifunctional bioplastics evaluated for mechanical, thermal, barrier, bioactive, and biodegradation properties.

## Materials and methods

### Materials and chemicals

Pea pods (*Pisum sativum* L.), bean pods (*Phaseolus vulgaris* L.), and fresh globe artichokes (*Cynara scolymus* L.) were sourced from local vegetable processing facilities located in the Ariana Governorate, Tunisia. The vegetable byproducts were transported to the laboratory in polyethylene bags and stored at 4 °C prior to processing.

Choline chloride (>98 %) and oxalic acid dihydrate (>99 %) DPPH (2,2-diphenyl-1-picrylhydrazyl), ABTS (2,2-Azino-bis (3-ethylbenzothiazo-line-6-sulphonic acid)) and Trolox (6-hydroxy-2,5,7,8-tetramethylchromane-2-carboxylic acid) were acquired from Sigma-Aldrich (Sigma-Aldrich, St. Louis, MO, USA).

### Pretreatment of vegetable waste by DES

Each type of vegetable waste was washed, dried overnight in a Labconco tray dryer at 50 °C, and then ground to a fine powder using a Polymix PX-MFC 90D mill (Kinematica AG, Malters, Switzerland) equipped with a 1  mm sieve. The processed vegetable powders were stored in amber glass jars at room temperature in the dark until needed. Briefly, the DES was prepared by mixing choline chloride and oxalic acid in a 1:1 M ratio. The mixture was heated at 80 °C under continuous magnetic stirring until a clear, homogeneous liquid formed. For biomass pretreatment, 1  g of vegetable powder was added to 10 g of DES (1:10 w/w), and the mixture was heated at 110 °C for 2  h under continuous stirring. This step promoted biomass dissolution and in situ lignin regeneration (Fig. 1), forming a viscous cellulose–lignin slurry [12].

**Fig. 1:**
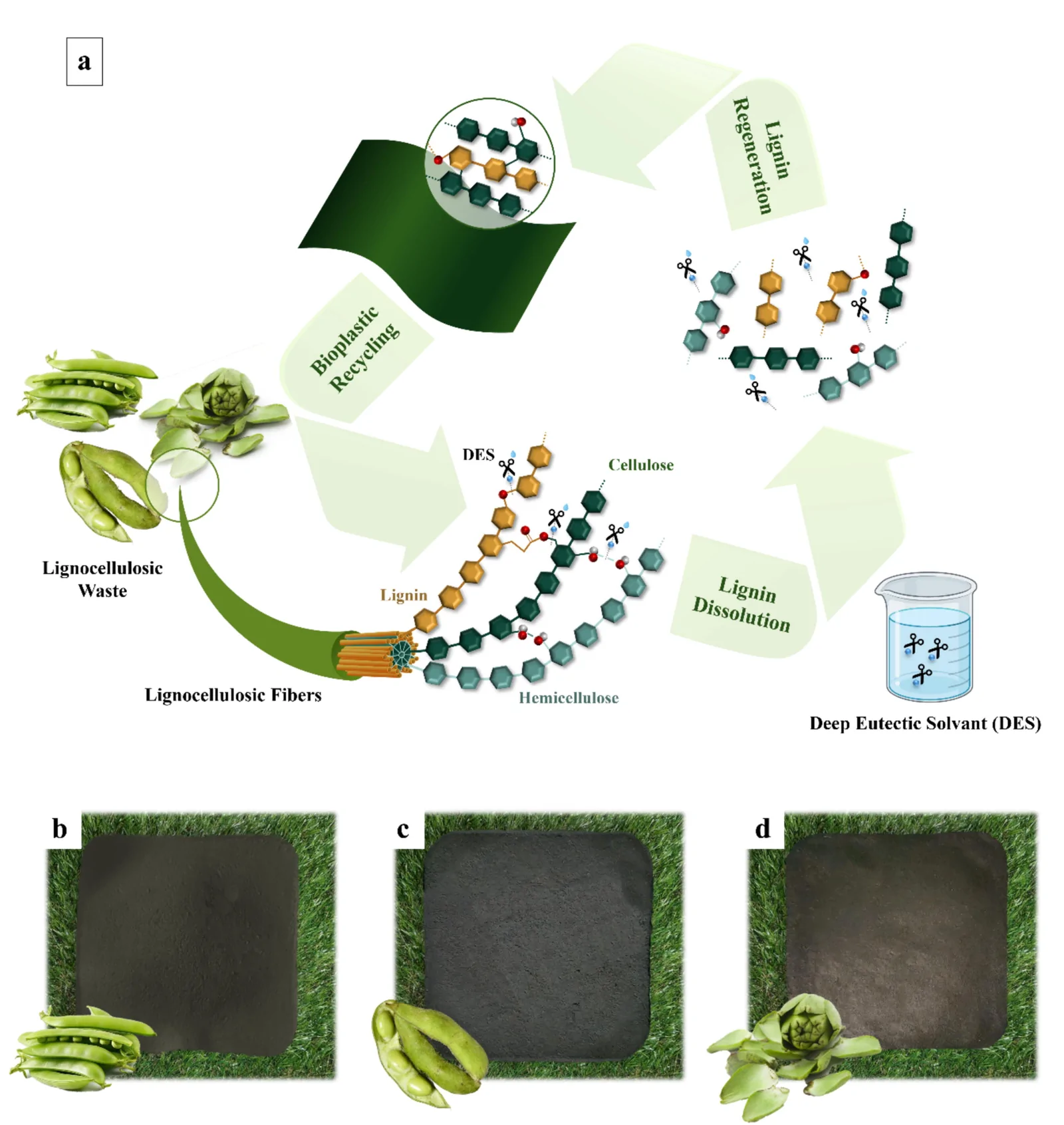
Schematic representation of DES-mediated lignocellulosic bioplastic and lignin regeneration process.

### Bioplastic fabrication

Firstly, the hot cellulose–lignin slurry was diluted with distilled water in a 1:10 (v/v) ratio to promote precipitation of the regenerated biopolymer network. The slurry was stirred for 2 h, followed by vacuum filtration. It was then subjected to successive washing with distilled water to remove residual solvent. This process yielded two important fractions: a solid phase intended for bioplastic fabrication and a liquid filtrate carrying the DES-water mixture. The solid phase containing cellulose–lignin complex was homogenized using an Ultra-Turrax homogenizer (IKA T25, IKA-Werke GmbH, Germany) at 10,000  rpm for 2  min to obtain a uniform slurry. Finally, the homogenized cellulose-lignin mixture (20 % solids) was cast into Teflon-coated Petri dishes (12  cm diameter) and dried at 60 °C for 24  h in a ventilated oven to form self-supporting bioplastic films [14]. After drying, films were carefully peeled off and conditioned at 23±2 °C and 50±5 % RH for at least 48  h prior to characterization. Bioplastic films have a thickness of 0.120±0.014 mm.

As for the liquid filtrate, it was collected for DES recovery by controlled evaporation of water at atmospheric pressure (70 °C). This condition was intentionally selected to preserve the chemical integrity and hydrogen-bonding structure of the oxalic acid-choline chloride DES, while ensuring effective water removal. The recovery yield of DES was quantified, showing that 94 % of the solvent mass was successfully recovered during the first cycle. The functional performance of the recovered DES was evaluated through its reuse in subsequent delignification cycles, and the solvent was successfully reused for up to five consecutive cycles, after which a noticeable decline in delignification efficiency and cellulose solubility was observed, primarily due to partial consumption of oxalic acid and the accumulation of lignin-derived impurities.

### Characterization and testing

#### Chemical composition of vegetable processing waste

The chemical constituents of the raw vegetable powders, including holocellulose, cellulose, lignin, and water and extractive contents, were determined following standardized protocols adapted from TAPPI [17,18]. Lipid extraction from the raw material was performed using the chloroform–methanol method as described by Burgos et al. [19]. The lipid content was quantified gravimetrically by weighing the dried lipid pellet and expressing it as a percentage of the initial dry weight of the sample. Protein extraction followed the protocol outlined by Deans et al. [20], and quantification was conducted using the Bradford colorimetric assay [21]. Soluble sugars were extracted according to the method of Gomez et al. [22], and their concentration was determined using the phenol–sulfuric acid assay adapted for microplate format [23].

#### Structural analysis

Structural features and chemical interactions were investigated using Fourier-transform infrared spectroscopy (FTIR) performed on a Bruker Equinox 55 instrument with ATR configuration. Spectra were acquired in the range of 4000 to 500 cm^−1^ with a resolution of 4 cm^−1^. Surface morphology was assessed by scanning electron microscopy (SEM) using a Zeiss EVO® LS 10, operated under high vacuum at an accelerating voltage of 15 kV, after sputter coating the samples with gold.

#### Molecular docking

To complement structural analyses, molecular docking was employed to investigate the molecular interactions between regenerated lignin and cellulose following DES treatment. It should be noted that the molecular docking approach employed in this study relies on simplified molecular models, including a short crystalline cellulose oligomer and representative lignin fragments. While these models do not capture the full amorphous, heterogeneous, and polymeric nature of cellulose and lignin after DES treatment, they are widely used to probe local interaction mechanisms at the molecular scale. Molecular docking simulations were performed using the Auto Dock 4.2 software package [24]. Chemically accurate structures derived from post-DES treatment profiles of lignin, cellulose, and oxalic acid were retrieved from the study of Xia et al. [13] and Maurya & Manik [25].

Cellulose backbone was constructed as a crystalline oligomer (cellotetraose), composed of β-(1 → 4)-linked D-glucopyranose units, to capture the hydrogen bonding potential inherent to its hydroxyl-rich backbone. It has been established that ChCl-oxalic acid DES preferentially disrupts amorphous cellulose regions while stabilizing short ordered domains, thereby justifying the use of a crystalline cellotetraose model to represent persistent hydroxyl-rich motifs following treatment [15]. Oxalic acid was covalently anchored or hydrogen-bonded to the cellulose surface at the C6 primary hydroxyl groups to stimulate plausible esterification or non-covalent interactions. Similarly, DES-induced lignin depolymerization exposes guaiacyl and syringyl units enriched in phenolic hydroxyl and methoxy groups, which are considered the dominant functional motifs governing hydrogen bonding and π-interactions with cellulose.

All the molecules were drawn using ACD (3D viewer) software (http://www.filefacts.com/acd3d-viewer-freeware-info). The molecular geometries of both lignin and cellulose were optimized, and the missing Gasteiger charges were added to the system during the preparation of the receptor input file. Next, AutoDock Tools were used for the preparation of all ligand and receptor files, where their PDB formats were converted into extended PDBQT (Protein Data Bank, Partial Charge, and Atom Type) format for docking analysis. Docking simulations were then conducted with AutoDock Vina, selected for its robustness in evaluating hydrogen bonding. The docking grid was centered on the cellulose backbone, encompassing regions rich in hydroxyl groups and oxalic acid to allow lignin fragments to explore binding near hydrogen donor and acceptor sites. The grid box dimensions were set at 50 Å × 12 Å × 12 Å with a grid spacing of 0.375 Å. Binding affinities were expressed in kcal/mol, and docked complexes were visualized using PyMOL 0.99rc6 [26] to annotate the hydrogen bonds.

#### Differential scanning calorimetry (DSC) analysis

Thermal behavior of the bioplastics was studied via DSC using a TA Instruments Q1000 unit, following the procedure outlined by Aloui and Khwaldia [27].

#### Water barrier properties

Moisture content (MC) and water solubility (WS) of the bioplastic films were determined following the method of Aloui et al. [28].

Water vapor permeability (WVP) was determined gravimetrically in triplicate following ISO 2528 [29]. Films were mounted on permeation cells containing silica gel and placed in a humidity chamber maintained at 38 °C and 90 % relative humidity; WVP was then calculated as follows:

$$\mathrm{WVP}\left(mm.m^{-2},day^{-1}.kPa^{-1}\right)=\frac{\mathrm{WVTR}\times T}{\Delta P}$$

where WVTR is the water vapor transmission rate, calculated as the slope of the weight gain/loss curve (g/d) normalized by the exposed film area (m^2^); T is film thickness (mm); ΔP is the water vapor partial pressure difference across the film (kPa).

#### Mechanical performance analysis

Mechanical performance, including tensile strength (TS), Young’s modulus (YM) and elongation at break (%E), was determined using an Instron 5965 universal testing machine under controlled conditions (23±2 °C, 50±5 % RH). Rectangular strips (15 mm × 100 mm) were tested in accordance with ISO 1924–2, as previously outlined by Aloui and Khwaldia [30], and results represent the mean of six measurements per sample.

#### Optical properties

Optical transmittance in the UV–visible range (200–800 nm) was recorded using a UV–Vis spectrophotometer (Drawell 722G, China).

#### Phenolic content determination

Total phenolic content (TPC) was measured using the Folin–Ciocalteu method according to Flores-Castañón et al. [31]. Gallic acid (GA) was used as a calibration standard (10–200 µg/ mL, R^2^ = 0.999). The absorbance of reduced phosphomolybdate/ tungstate complex, proportional to total phenolics, was monitored at 725 nm (FlexA-200 microplate reader, ALLSHENG Instrument Co., Ltd., Hangzhou, China). Results were expressed as mg of GA equivalents per g of dry weight (mg GAE/g DW). Total flavonoid content (TFC) was determined according to the AlCl_3_ colorimetric method [32]. The absorbance of samples at 430 nm, as compared to quercetin standard solution (20–200 µg/mL, R^2^ = 0.993), was used to calculate the TFC, expressed as quercetin equivalent (mg QE/ g DW).

#### Antioxidant capacities

Using the methods described by Khwaldia et al. [33], the antioxidant activity of the bioplastic films was evaluated in vitro using DPPH (1,1-diphenyl-2-picrylhydrazyl), ABTS (2,2′-azino-bis3-ethylbenzothiazoline-6-sulfonic acid) free radical scavenging activity, and FRAP assays (ferric reducing antioxidant power). For each antioxidant assay, a precisely weighed sample of bioplastic film or an aliquot of the vegetable powder was extracted by maceration in ethanol 50 % for 24 h. After centrifugation, the supernatant was incubated with its respective reagent at room temperature for 30 min. Following incubation, the absorbance was determined at specific wavelengths for each assay: 515 nm (DPPH), 595 nm (FRAP), and 734 nm (ABTS) using a FlexA-200 microplate reader. Results were expressed in milligrams of Trolox equivalents per gram of sample (mg TE/g).

#### Antimicrobial activities

The antimicrobial properties were assessed via the agar well diffusion method against five microbial strains: *Escherichia coli*, *Salmonella Typhimurium*, *Staphylococcus aureus*, *Enterococcus faecalis*, and *Candida albicans*. Zones of inhibition were measured following a previously established protocol [34] and compared to gentamicin as a positive control.

#### Biodegradability evaluation

Biodegradation capacity was evaluated by burying film specimens (10 × 10 mm) at a depth of 5 cm in aerated, non-sterile soil collected from the Sidi Thabet perimeter [35]. The soil had an alkaline pH of 8.66±0.05 and was biologically active, containing native microbial communities that ensured biodegradation activity. Soil moisture was maintained at 50 % of its water holding capacity by periodic addition of 3 ml of water to sustain microbial viability. Tha samples were incubated at ambient temperature under aerobic conditions to mimic natural environmental conditions and ensure realistic soil degradation behavior. Film specimens were retrieved after 10, 20, 30, 40, 50, 60 and 70 days, cleaned, dried at 70 °C for 24 h, and weighed to determine mass loss. Biodegradation was expressed as the percentage reduction in weight over time, and tests were carried out in triplicate.

#### Statistical analysis

All experiments were conducted in at least triplicate. Data were expressed as mean±standard deviation (SD). Statistical analysis was performed using SPSS 22.0 software (IBM Corp., Armonk, NY, USA). Where significant differences were observed (P ≤0.05), Tukey HSD test was used for post hoc comparison among group means.

## Results and discussion

### Composition of vegetable waste powders

The initial characterization of the raw vegetable waste powders revealed distinct differences in lignocellulosic composition, which are likely to influence the physicochemical and mechanical properties of the resulting bioplastic films. As shown in Table 1, artichoke bracts exhibited the highest α-cellulose content (31.09±0.63 %), followed by bean pods (21.17±0.72 %) and pea pods (20.39±0.53 %). Lignin content was also highest in artichoke bracts (18.34±0.95 %), whereas pea pods and bean pods contained 10.49±0.99 % and 6.39±0.44 %, respectively. In contrast, hemicellulose levels were greater in the legume pods, with bean pods containing 19.26±1.01 % and pea pods 15.81±0.68 %, compared to 15.13±0.74 % in artichoke bracts. These compositional differences directly motivated our hypothesis that the intrinsic lignocellulosic heterogeneity of the raw materials would translate into distinct structural, mechanical, and functional properties in the resulting bioplastic films.

**Table 1:** Chemical composition of raw materials (wt%).

	Pea pods	Bean pods	Artichoke bracts
Water soluble compounds	40.27±0.41^c^	37.84±0.95^b^	26.80±0.21^a^
Extractives	5.90±0.36^c^	2.94±0.89^a^	4.55±0.11^b^
Lignin	10.49±0.99^b^	6.39±0.44^a^	18.34±0.95^c^
Cellulose	20.39±0.53^a^	21.17±0.72^a^	31.09±0.63^b^
Hemicellulose	15.81±0.68^a^	19.26±1.01^b^	15.13±0.74^a^
Ash	5.35±0.17^a^	7.75±0.79^b^	7.23±0.19^b^
Soluble proteins	3.07±0.47^a^	3.19±0.54^a^	2.77±0.32^a^
Lipids	0.91±0.04^a^	1.14±0.06^b^	1.29±0.12^c^
Soluble sugars	20.57±0.21^b^	26.82±0.33^c^	0.72±0.03^a^
Moisture	8.53±0.13^b^	9.15±0.05^c^	7.30±0.05^a^

These results are generally consistent with literature values for similar vegetable residues, although variations are expected due to differences in cultivar, cultivation conditions, harvest stage, plant part analyzed, and analytical methodology. In particular, the α-cellulose content determined for artichoke bracts in this study (31.09±0.63 %) falls within the lower bound of reported ranges for *Cynara scolymus* residues (30–50 %) [36]. However, the hemicellulose content measured for artichoke bracts in this study (15.13±0.74 %) is lower than literature values commonly reported for artichoke leaves/stalks (20–40 %), whereas the lignin content (18.34±0.95 %) is higher than the 5–15 % range frequently cited [36]. These variations may reflect differences in the sampled tissue (leaves, mixed aerial parts), the presence of non-lignin phenolics counted in certain lignin assays, or methodological differences.

In contrast to cereal residues such as rice straw, wheat straw, corn stover, and sugarcane bagasse, which generally contain higher cellulose fractions (33–46 % cellulose), the cellulose contents of pea and bean pods in this study (20–21 %) are much lower [37]. Their hemicellulose and lignin contents reflect the compositional signature of legume pods rather than that of lignocellulosic straws or bagasse (reported ranges 18–35 % hemicellulose, and 7–29 % lignin) [37].

Comparing with specific studies, bean pod values are lower in cellulose and hemicellulose than those reported by Santos et al. (2020) [38] for Caupi bean pods (29.97 % cellulose; 24.33 % hemicellulose; 7.31 % lignin), whereas the lignin content is of comparable magnitude. Similarly, Bhiri et al. (2022) [39] reported higher α-cellulose (27.3±0.70 %) and hemicellulose (17.3±1.20 %) for pea pods than the levels obtained in the present work. In contrast, the lignin content measured in pea pods (10.49±0.99 %) is relatively close to that reported by Bhiri et al. (2022) [39]. The remaining discrepancies may stem from varietal/genetic differences, agro-climatic conditions, harvest maturity, and differences in extraction/quantification methods.

### Structural characterization of vegetable waste and bioplastic films

FTIR spectroscopy was employed to elucidate the chemical transformations induced by DES treatment on lignocellulosic agricultural residues, specifically bean pods, pea pods, and artichoke bracts, used as feedstock for bioplastic generation (Fig. 1b–d). The comparative spectra between raw biomass and their corresponding DES-treated bioplastics revealed distinct modifications in functional group regions, confirming both lignin regeneration and cellulose rearrangement (Fig. 2a–c). A broad absorption band around 3300 cm^−1^, attributed to O—H stretching vibrations, was intensified in all bioplastic samples, indicating enhanced hydrogen bonding and increased hydroxyl group exposure. This enhancement reflects cellulose reorganization and partial hydrolysis of amorphous regions by oxalic acid [12]. Importantly, the DES system may also introduce new hydrogen-bonding interactions between residual oxalic acid moieties and hydroxyl groups in cellulose, which could manifest as subtle shifts or broadening in the 3300 cm^−1^ region [40]. Peak at ∼2920 cm^−1^, corresponding to C—H stretching of methyl and methylene groups, persisted post-treatment, suggesting partial retention and possible condensation of lignin structures [40]. The band near 1720 cm^−1^, typically associated with C=O stretching in ester and carboxylic groups from hemicellulose and lignin-derived esters, was diminished or shifted, implying cleavage of ester linkages and selective hemicellulose removal by DES [41]. A sharper peak at ∼ 620 cm^−1^, often linked to conjugated C=C stretching or adsorbed water, was observed in pea pod and bean pod bioplastics, suggesting aromatic ring regeneration and increased hydrophilicity. Additionally, weak shoulders near 1420–1500 cm^−1^ may correspond to asymmetric COO^−^ stretching from residual oxalic acid interactions with cellulose hydroxyls, forming new hydrogen-bonded complexes that subtly shift the vibrational landscape [41]. Another notable peak around 1320 cm^−1^ corresponds to CH_2_ wagging vibrations in crystalline cellulose, and its increased intensity in treated samples reflects improved cellulose ordering and reduced amorphous content. This enhancement is consistent with the DES’s ability to disrupt native hydrogen bonding while promoting recrystallization of cellulose chains [42]. A distinct peak around 1200 cm^−1^, typically attributed to C–O stretching vibrations in secondary alcohols and ether linkages of cellulose and hemicellulose, was present in all bioplastic samples. Its persistence and sharpening suggest that while oxalic acid selectively hydrolyzes amorphous hemicellulose, it preserves or reorganizes cellulose ether bonds, enhancing the structural integrity of the polysaccharide backbone [41]. The band around 1040 cm^−1^, assigned to C—O—C and C—O—H stretching in cellulose and hemicellulose, was more defined in bioplastics, indicating improved cellulose ordering and crystallinity. In the lower fingerprint region (500–1000 cm^−1^), additional structural insights were revealed. Peaks between 600 and 900 cm^−1^ are typically associated with aromatic ring deformations and C–H out-of-plane bending, particularly from guaiacyl and syringyl units in lignin [41]. The presence and sharpening of these bands in DES-treated samples suggest partial lignin regeneration and structural condensation. Furthermore, bands around 890 cm^−1^ are indicative of β-glycosidic linkages in cellulose, and their increased intensity reflects enhanced cellulose crystallinity and reduced amorphous content [40]. These fingerprint region features confirm that DES treatment not only modifies major functional groups but also restructures the polysaccharide backbone and aromatic domains at a molecular level.

**Fig. 2:**
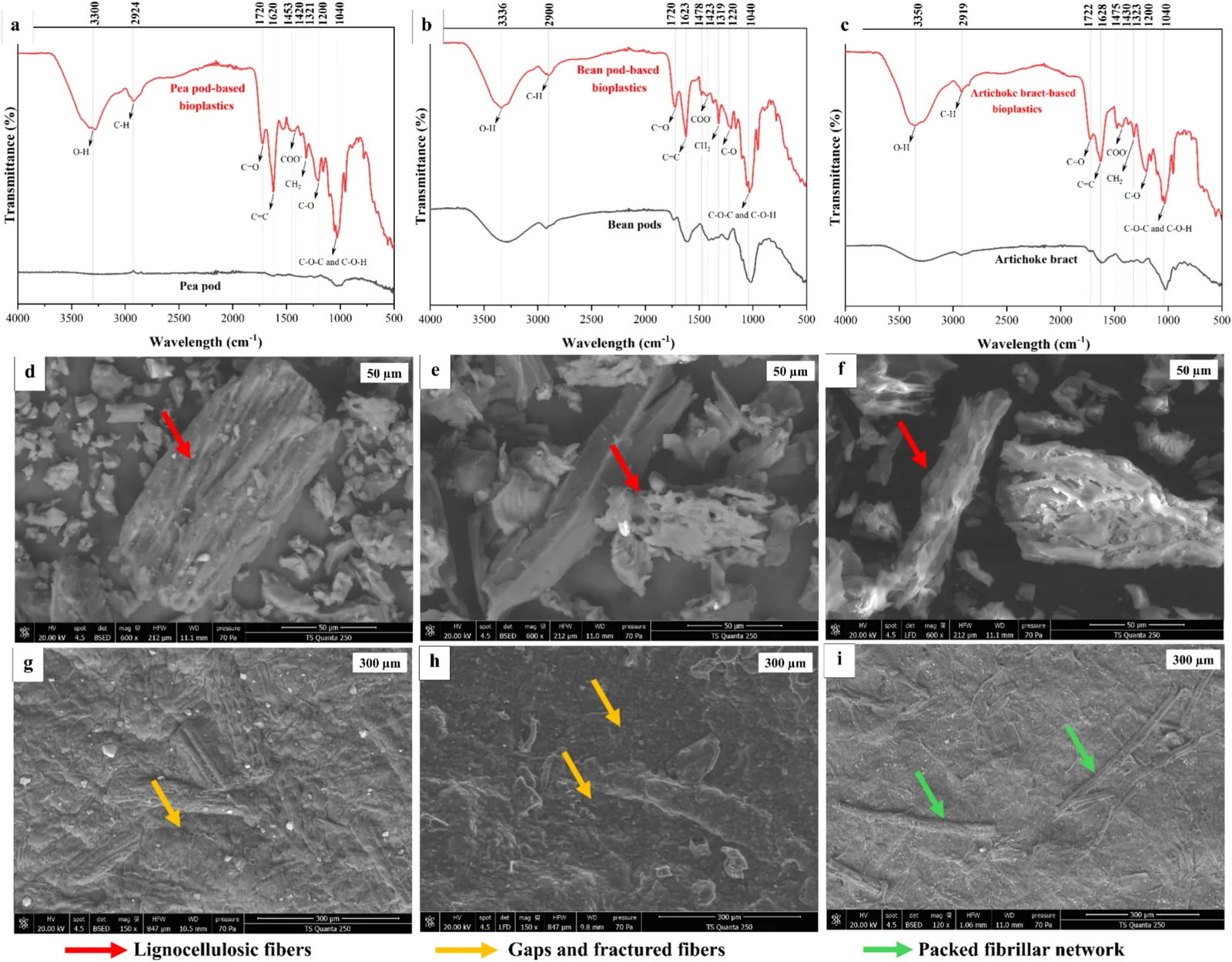
Visual, FTIR, and SEM characterization of pea pod-, bean pod-, and artichoke bract–based bioplastics and their raw materials: (a–c) Photographs of the generated bioplastics and their corresponding raw materials; (d–f) FTIR spectra comparing raw materials and bioplastics; (g–i) SEM micrographs of pea pods, bean pods and artichoke bracts powders, respectively; (j–l) SEM micrographs of the corresponding pea pod-, bean pod- and artichoke bract-based bioplastics.

These spectral changes align with previous reports on DES-mediated biomass fractionation, where selective solubilization of lignin and hemicellulose enhances the accessibility and structural integrity of cellulose, thereby improving the mechanical and barrier properties of the resulting bioplastics [40]. The distinct spectral profiles of each biomass type further reflect the inherent compositional differences and their unique responses to DES treatment, with artichoke bracts showing pronounced cellulose reorganization, pea pods retaining aromatic lignin features, and bean pods exhibiting enhanced hydrogen bonding and crystallinity. Collectively, the FTIR analysis confirms that DES treatment effectively restructures lignocellulosic matrices, enabling the valorization of agri-food waste into functional bioplastics with tailored chemical architectures.

SEM was employed to examine both the raw lignocellulosic materials and their corresponding DES-treated bioplastics derived from artichoke bracts, pea pods, and bean pods (Fig. 2). SEM micrographs of the raw and processed materials revealed clear structural transformations induced by oxalic acid–choline chloride DES treatment. Raw samples (Fig. 2d–f) exhibited elongated, loosely bound fibrils with visible surface roughness and fragmentation. The red arrows highlight individual lignocellulosic fibers and irregular fiber bundles, suggesting a heterogeneous distribution of structural polymers, which appear unorganized and discontinuous, typical of untreated plant matrices rich in amorphous hemicellulose and unstructured lignin.

Upon DES treatment, these matrices underwent significant reorganization, with each biomass responding uniquely based on its native composition.

Artichoke-based films (Fig. 2i) displayed a dense and homogeneous microstructure, characterized by aligned and tightly packed fibrillar networks (marked by green arrows), minimal surface voids, and smooth topography. This effective matrix consolidation is likely due to the high cellulose and lignin contents of artichoke bracts, which provides both structural reinforcement and cohesive matrix formation during DES treatment. The absence of voids and the uniform texture suggest strong inter-fibrillar bonding, consistent with DES-induced reorganization and lignin redistribution. Minichiello et al. [43] reported that artichoke bracts contain substantial amounts of crystalline cellulose and polyphenolic-rich lignin, which contribute to robust film architecture and reduced porosity. The DES system facilitates lignin redistribution and cellulose rearrangement, yielding a consolidated bioplastic surface.

In contrast, bean pod-based films (Fig. 2h) exhibited a more porous and heterogeneous morphology, with visible microvoids, irregular fibril alignment, and surface discontinuities (highlighted by yellow arrows). The images clearly showcase that the fibers are loosely packed, and the surface is interrupted by gaps and discontinuities. These features reflect the lower lignin and cellulose contents of bean pods compared to artichoke bracts, resulting in weaker matrix cohesion and less effective fiber entanglement. Although bean pods possess moderate cellulose levels, their reduced lignin concentration limits structural integrity during film formation. Murgia et al. [44] demonstrated that high pod shattering in common beans correlates with diminished lignin and cellulose content, which compromises mechanical strength and contributes to increased porosity in derived materials.

Pea pod-based films (Fig. 2g) presented an intermediate morphology, with moderate porosity and partial fibrillar alignment. The yellow arrows point to regions of fibrillar continuity, suggesting that pea pods support better matrix integrity. This transitional structure reflects the higher lignin content of pea pods relative to bean pods, enabling improved matrix cohesion while still exhibiting some heterogeneity which makes it not as robust as artichoke-derived bioplastic. The raw pea pod SEM images showed more compact fiber bundles than bean pods, and this translated into smoother bioplastic surfaces post-DES treatment. These compositional gradients across the three biomass types directly influenced the microstructural outcomes of DES processing, as the solvent system interacts differently with each matrix depending on its lignin-cellulose ratio and fiber architecture.

Overall, the SEM analysis confirms the effect of DES on lignocellulosic and highlights that the microstructural integrity of DES-derived bioplastics is governed by the native biochemical composition of the feedstock, particularly the relative abundance of cellulose and lignin.

### Docking analysis of reassembled lignin and cellulose: Mapping affinity in bioplastic architectures

The structural transformations revealed by FTIR, such as enhanced hydrogen bonding and cellulose crystallinity, alongside the microstructural patterns observed via SEM, are further substantiated at the molecular level through docking simulations, which illuminate the specific non-covalent interactions between regenerated lignin and cellulose that underpin the film’s structure and functional integrity. This computational approach predicts energetically favorable binding conformations, offering insight into the molecular affinities that govern matrix cohesion. Specifically, regenerated lignin, enriched in phenolic and aromatic domains, interfaces with cellulose hydroxyl groups through several interactions and especially hydrogen bonding, all of which contribute to stabilizing the biopolymer network [45]. These interactions have been widely documented in lignocellulosic systems, where hydrogen bonds between lignin hydroxyls and cellulose oxygen atoms play a dominant role in adhesion, while aromatic stacking and van der Waals forces enhance interfacial compatibility [46,47]. In the context of DES-mediated reorganization, such molecular-level affinities are critical for understanding how lignin reintegration reinforces the structural integrity of the bioplastic. Docking analysis thus serves as a bridge between experimental morphology and molecular design, illuminating the cohesive forces that underpin sustainable film formation.

This study successfully visualized, for the first time, the molecular interaction between regenerated lignin and cellulose using docking simulations (Fig. 3a). The regenerated lignin post-DES treatment exhibited binding energies of −4.9 and −5.2 kcal/mol, indicating moderately stable non-covalent interactions (Fig. 3c). These results provide a novel mechanistic perspective, since previous research largely emphasized macroscopic film properties without considering molecular-level binding. Docking poses revealed dominant hydrogen bonding between lignin’s aromatic rings and cellulose’s hydroxyl-rich surface, suggesting a synergistic interface capable of enhancing film mechanical integrity (Fig. 3b). Hydrogen bonding played a central role in stabilizing the complexes, with phenolic hydroxyls of lignin forming up to four hydrogen bonds in complex 1 and two in complex 2 with the C2 and C3 hydroxyl groups of cellulose (Fig. 3d). Oxalic acid, pre-functionalized on cellulose, enhanced these interactions by introducing carboxyl-rich anchoring sites. Docking simulations revealed up to two hydrogen bonds in complex 1 and one in complex 2 (Fig. 3d), consistent with oxalic acid’s bifunctional carboxyl groups acting as a crosslinking agent capable of interacting with both cellulose hydroxyls and lignin phenolics [48,49]. This pre-functionalization step strengthens the anchoring of regenerated lignin units, which are known to engage in hydrogen bonding with modified polysaccharide matrices [50]. The observed interactions are consistent with prior reports highlighting oxalic acid’s role in modulating lignocellulosic structure [13]. Specifically, oxalic acid influences cellulose crystallinity, modifies surface energy, and facilitates lignin rearrangement, thereby improving cellulose-lignin compatibility [51]. This dual role of oxalic acid, as a structural modifier and an interaction mediator, aligns with its ability to alter surface energy and crystallinity [52]. Collectively, these results support the hypothesis that regenerated lignin, when interfaced with oxalic acid-modified cellulose, forms a structurally coherent and functionally robust bioplastic network.

**Fig. 3:**
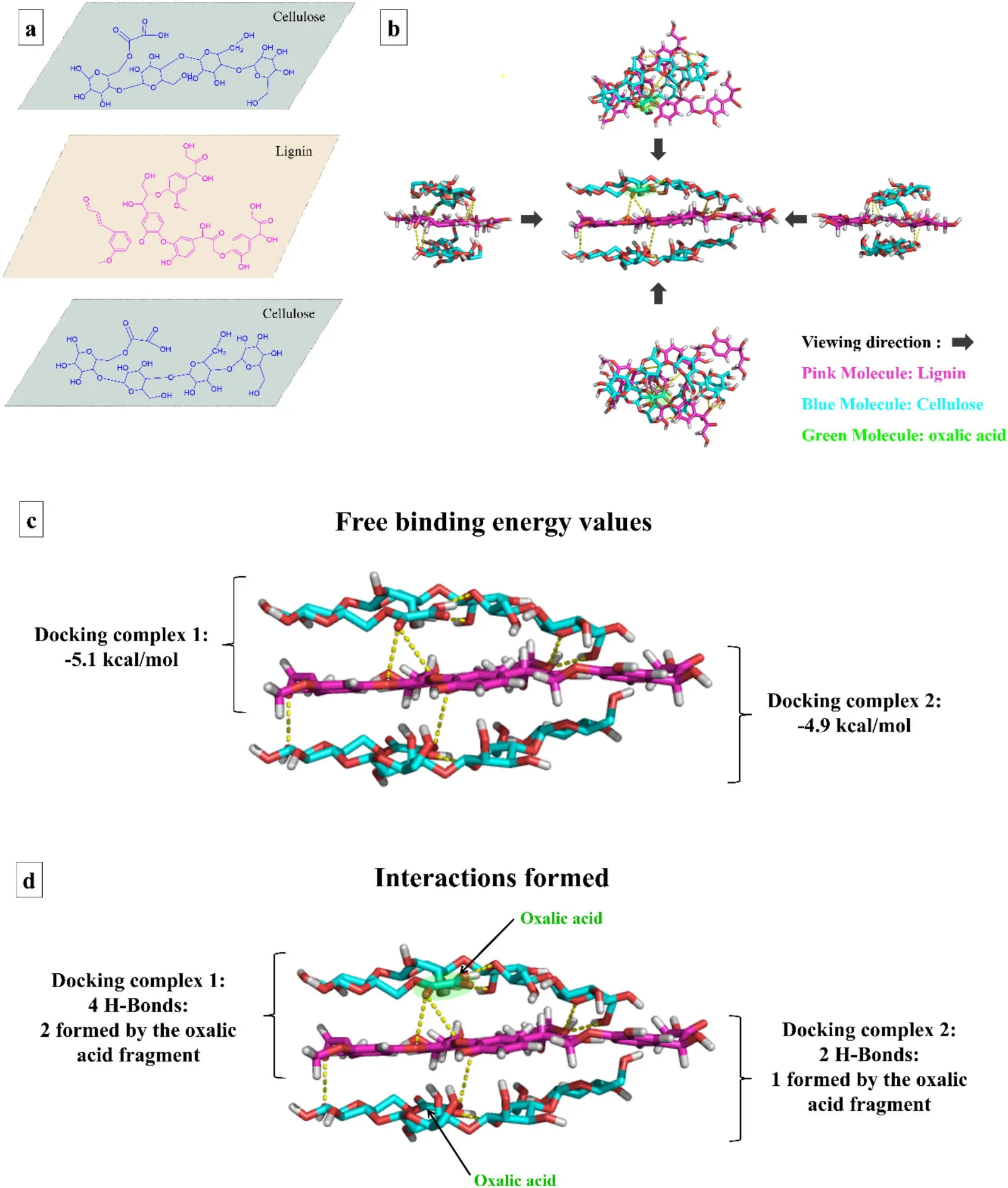
Molecular Docking analysis of structural interaction of DES-regenerated bioplastic components. (a) Simulated post-treatment structure of regenerated bioplastic matrix following DES application; (b) Molecular docking interactions between regenerated lignin and cellulose, visualized from four spatial orientations; (c) Binding energy values of Complex I (cellulose-lignin) and Complex II (lignin-cellulose) derived from docking simulations; (d) Quantitative analysis of hydrogen bonding and oxalic acid contributions within each molecular complex.

The DES treatment further enhanced polarity and conformational flexibility of regenerated lignin, which facilitated its binding affinity to cellulose. The docking results suggest that regenerated lignin not only associates effectively with cellulose but also contributes to a non-covalent, cooperative molecular architecture that may enhance barrier properties and bioactivity. This molecular synergy corroborates experimental findings of improved physico-mechanical and biological properties, as lignin retained its antioxidant or antimicrobial functionalities after regeneration.

### Mechanical properties of bioplastics

The tensile strength (TS), Young’s modulus (YM), and elongation at break (%E) of the developed bioplastic films are presented in Table 2. Significant differences (P < 0.05) were observed among the three formulations. Artichoke bract-based bioplastics exhibited the highest TS (7.02±0.68 MPa) and YM (428.11±44.47 MPa), indicating superior stiffness and resistance to deformation. In contrast, bean pod-based films displayed the greatest elongation at break (8.35±1.30 %), suggesting enhanced flexibility, while pea pod-based films demonstrated intermediate mechanical performance.

**Table 2:** Mechanical and water barrier properties of bioplastic films.

	Pea pod-based bioplastic	Bean pod-based bioplastic	Artichoke bract-based bioplastic
MC (%)	12.35±0.85^a^	16.82±0.49^c^	14.06±0.33^b^
WS (%)	26.01±1.14^a^	43.93±0.29^c^	33.86±0.09^b^
WVP (g.mm/m^2^.d.kPa)	0.037±0.001^a^	0.063±0.004^c^	0.046±0.004^b^
TS (MPa)	4.05±0.48^a^	4.56±1.27^a^	7.02±0.68^b^
E (%)	4.19±0.54^a^	8.35±1.30^b^	3.34±0.63^a^
YM (MPa)	242.81±43.69^b^	164.68±69.20^a^	428.11±44.47^c^

All values are presented as means±SD. Different letters are displayed in a column indicate significant differences (P < 0.05). TS, tensile strength; E, elongation at break; YM, Young's Modulus; MC, moisture content; WS, water solubility; WVP, water vapor permeability.

These variations can be directly linked to the lignocellulosic composition of the raw materials. Artichoke bracts contained the highest α-cellulose content (31.09±0.63 %), which is known to enhance intermolecular hydrogen bonding and polymer chain alignment within the matrix, resulting in improved mechanical strength and rigidity [53]. Conversely, the higher hemicellulose content of bean pods (19.26±1.01 %) likely contributed to their lower YM and greater extensibility, as hemicelluloses disrupt tight cellulose packing and increase amorphous regions, promoting flexibility [53]. The lignin content also appears to play a role. Bean pod-based bioplastics, despite their lower TS compared to artichoke bract films, contained the lowest lignin proportion (6.39±0.44 %). This low lignin content likely reduces matrix rigidity but, when combined with their high hemicellulose content (19.26±1.01 %), favors flexibility and chain mobility, explaining the highest elongation at break observed (E = 8.35±1.30 %). Lignin’s aromatic and hydrophobic nature can act as a rigid filler, but its lower content results in softer and more ductile films. In contrast, the higher lignin content in pea pods (10.49±0.99 %) likely contributes to their lower extensibility (%E = 4.19±0.54 %) by increasing stiffness and limiting chain slippage during deformation. SEM micrographs supported these findings, revealing that artichoke bract-based films possessed a denser and more homogeneous microstructure (Fig. 2i), while bean pod-based films displayed a more porous and heterogeneous morphology (Fig. 2h), which correlates with their lower YM but higher %E.

Overall, our results align with previous studies on lignocellulose-based films, which consistently report that high cellulose content enhances strength and modulus, while increased hemicellulose promotes flexibility [53]. The interplay between cellulose crystallinity, hemicellulose plasticizing effects, and lignin reinforcement appears to be a key determinant of the mechanical performance of agro-waste-derived bioplastics [54].

When benchmarked against other agro-waste-derived bioplastics, the tensile properties of the present vegetable processing waste bioplastics are comparable to those of low-to-moderate strength packaging materials intended for flexible or semi-rigid applications (e.g., dry food wraps, inner liners, sachets, and short-shelf-life protective films). Starch or peel-based films typically exhibit very low TS (<1 MPa) and limited stiffness, primarily due to their highly amorphous structure and reliance on plasticizers [55,56]. In contrast, pectin-rich citrus peel films demonstrate substantially higher strength values (5–45 MPa), although this improvement is often accompanied by reduced %E [57]. Regenerated lignocellulosic bioplastics obtained from wood or bamboo leaves via DES processing achieve TS exceeding 100 MPa, 113 and 128 MPa, respectively [13,14]. The intermediate mechanical performance achieved by the present bioplastics reflects a deliberate trade-off between functional adequacy and processing sustainability, supporting the suitability of these materials for disposable, biodegradable packaging rather than rigid structural applications.

### Water barrier properties of bioplastics

The water barrier properties of bioplastics derived from vegetable waste, assessed in terms of moisture content (MC), water solubility (WS), and water vapor permeability (WVP), are presented in Table 2. Pea-pod films exhibited the lowest MC (12.35±0.85 %), followed by artichoke-based films (14.06±0.33 %) and bean-pod films (16.83±0.50 %). Lower film MC indicates a less hydrophilic nature and a more compact structure, which reduces the free volume available for water vapor diffusion. This result is consistent with SEM observations, which revealed a compact and uniform microstructure in artichoke-based bioplastic (Fig. 2i), in contrast to the more porous and heterogeneous architecture observed in bean-pod bioplastic (Fig. 2h). Water retention and vapor diffusion are governed by polymer matrix hydrophilicity and density [58]. Bean-pod films had the highest WS (43.93±0.29 %), followed by artichoke-based films (33.86±0.09 %) and pea-pod films (26.02±1.14 %) (Table 2). This can be attributed to the more open structure of bean-pod bioplastics, which facilitates water penetration and dissolution of soluble fractions, whereas the compact networks in artichoke and pea-pod films may decrease water access to hydrophilic domains. Similar correlations between porosity and WS have been reported for biodegradable polyesters and chitosan-based composites [59,60].

The WVP values confirmed the trends observed for WC and WS, with pea-pod films showing the highest water vapor barrier properties (0.037±0.001 g·mm m^−2^ day^−1^ kPa^−1^), followed by artichoke (0.046±0.004 g·mm·m^−2^ day^−1^ kPa^−1^), and bean-pod (0.063±0.004 g·mm m^−2^ day^−1^ kPa^−1^). With a relatively compact structure, pea-pod films offered the most tortuous diffusion pathways, whereas porous bean-pod films provided shorter paths for vapor transport. This is in line with well-established microstructure–transport relationships in biopolymer films [58]. It has been established that DES treatment followed by in situ lignin regeneration is expected to enhance barrier properties by promoting controlled disassembly and reassembly of fibrils into compact matrices and redistributing lignin as a cohesive, relatively hydrophobic phase. Thereby decreasing free volume and reducing overall polarity, improving water-barrier performance. Similar effects have been observed by Moon et al. [61] in DES-treated cellulose/lignin materials, which form denser morphologies with reduced WVP compared with untreated systems. Moreover, lignin is known to contribute to intrinsic hydrophobicity and UV-shielding due to its aromatic structure [58].

Overall, pea-pod films provided the highest water barrier performance, combining the lowest MC, WS, and WVP. Artichoke films exhibited intermediate performance, whereas bean-pod films, characterized by higher porosity, displayed the highest solubility and permeability. The close relationship between microstructure and property is in line with diffusion-based transport models and SEM–barrier correlations reported for biopolymer films [62].

### DSC analysis and thermal behavior

DSC thermograms of vegetable processing waste and derived bioplastics were shown in Fig. 4. The thermal degradation temperatures (T_d_) of the vegetable waste powders and their corresponding bioplastics highlight significant improvements in thermal stability, particularly in the bioplastic films. For instance, the artichoke bract waste powder had the highest T_d_ (237.9 °C), reflecting its greater thermal stability due to higher cellulose and lignin contents. This thermal stability was further enhanced in the artichoke bract-based bioplastic, which exhibited an even higher T_d_ of 266.2 °C, indicating improved resistance to thermal degradation. Similarly, both pea pod- and bean pod-based bioplastics showed improved T_d_ values (243.9 °C and 244.2 °C) compared to their corresponding raw powders (191.4 °C and 174 °C, respectively). These main endothermic transitions are associated with lignin decomposition and the disruption of ordered cellulose domains within the lignocellulosic matrix. In addition, the pea pod-based bioplastic exhibited a distinct thermal event at approximately 190°C, which can be attributed to an early onset of thermal degradation, corresponding to the initial softening and partial depolymerization of lignin and the disruption of less ordered cellulose domains. The greater prominence of this feature in pea pod-based bioplastics reflects differences in lignocellulosic composition and the relative distribution and interactions of lignin and cellulose in this feedstock.

**Fig. 4:**
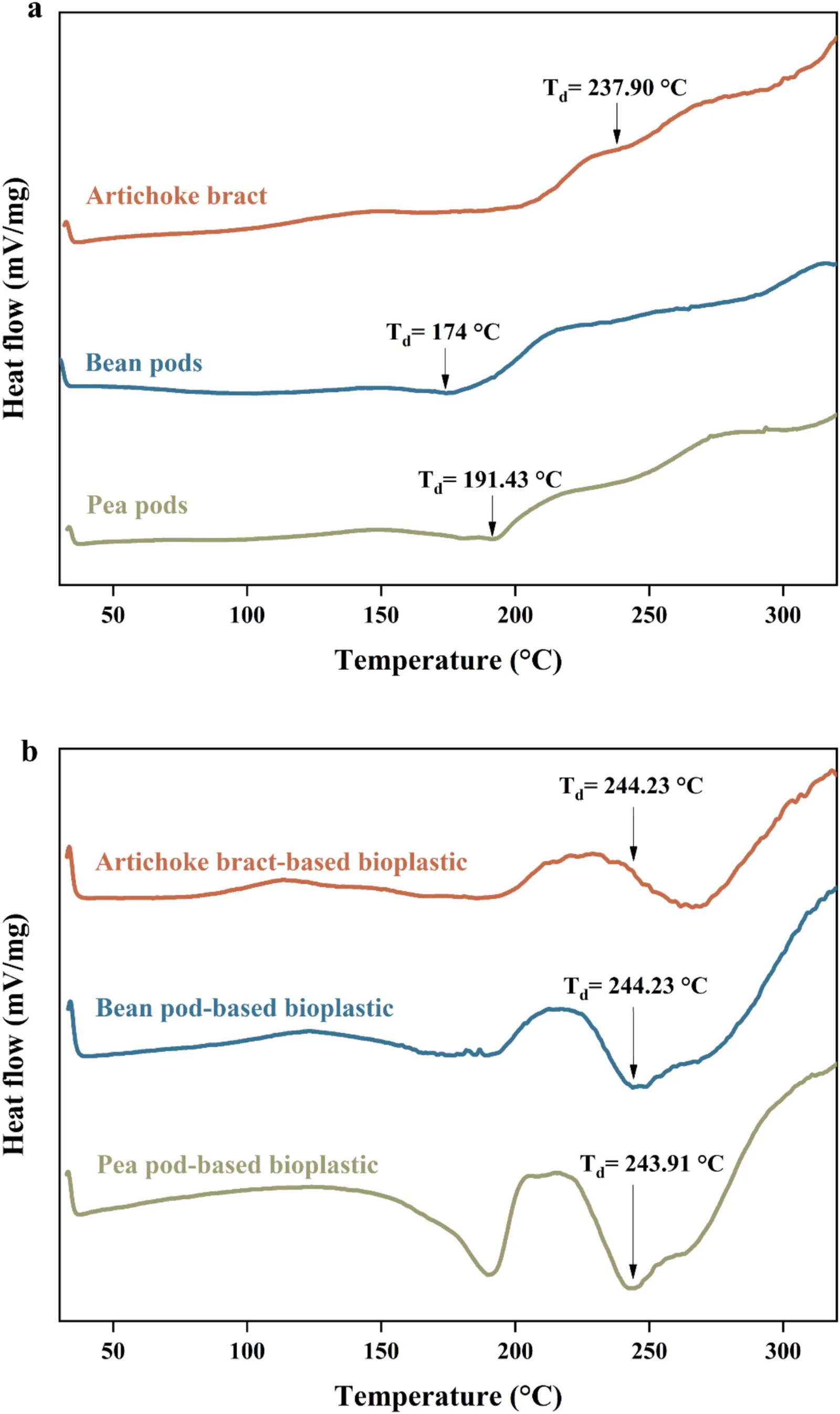
DSC thermograms of (a) vegetable processing wastes and (b) their corresponding bioplastics.

Overall, the improved thermal stability of the bioplastics can be attributed to DES-induced lignin reorganization, partial depolymerization, and enhanced intermolecular interactions within the regenerated lignocellulosic matrix, which delay thermal degradation. This trend aligns with the findings of Chandra et al. [63], who reported degradation temperatures around 257 °C for bioplastics made from lignocellulosic plant-based resources. These results confirm that the DES treatment not only facilitates lignin extraction but also enhances the thermal performance of the bioplastics.

### Light barrier properties of bioplastics

The UV–Vis spectra (200–800 nm) show that all bioplastic films transmit <2 % light across the measured range, indicating exceptional light-blocking capacity. Such performance is consistent with the presence of lignin-rich lignocellulosic matrices, as lignin’s aromatic phenylpropanoid units (π–π and n–π* transitions) strongly absorb UV light, enabling near-complete screening of UVA/UVB even at low loadings in cellulose-based films. Sadeghifar and Ragauskas (2020) [64] demonstrated that adding only ∼2 wt% lignin to cellulose can block 100 % of UV-B (280–320 nm) and >90 % of UV-A (320–400 nm), underscoring the intrinsic UV-shielding role of lignin in biopolymer matrices.

Compared with other agro-waste and biopolymer films, our films exhibit a total transmittance <2 %, significantly lower than that of comparable agro-waste and biopolymer films. Starch-based films typically require chemical modification or additives to achieve effective UV barriers; even then, shielding is often significant only in the UV region rather than across the whole UV–visible spectrum [60]. Pectin-based films derived from citrus wastes can reduce UV transmittance and, in some formulations with nanoemulsions, reach near-zero transmission at 280 nm, but they commonly display higher transmittance in parts of the visible region compared with the present lignocellulosic films [65]. From an application perspective, strong UV/visible light blocking is advantageous for packaging photosensitive foods (e.g., products rich in riboflavin, chlorophylls, or unsaturated lipids), where photooxidation compromises quality and shelf life. The combination of very low transmittance and bio-sourced composition therefore positions these vegetable-waste-derived bioplastics as promising light-barrier materials for active and protective food packaging, with performance that meets or surpasses many current agro-waste reported in the literature.

### Antioxidant activity

The results of phenolic content and antioxidant capacities of the prepared bioplastics, as well as the raw vegetal waste (artichoke bracts, pea pods, and bean pods) and their DES extracts, are reported in Table 3. The raw powders extracted with 50 % ethanol showed a moderate phenolic content corresponding mainly to the free phenolic fraction. The highest TPC was obtained with bean pods (26.9 mg GAE/g), corresponding to the strongest antiradical scavenging capacities (DPPH: 209 mg TE/g, ABTS: 62.9 mg TE/g). This observation is consistent with the findings of Fendri et al. [66], who reported that the phenolic content of bean pods (*Phaseolus vulgaris*) exceeds that of pea pods (*Pisum sativum*) across different extraction solvents. DES substantially increased TPC in all samples, particularly pea pods (60 mg GAE/g). This effect indicates the DES superior solubilization potential for both polar (phenolic acids) and moderately non-polar antioxidants (flavonoid aglycones), highlighting its potential to disrupt hydrogen bonding and release bound phenolics from plant cell walls [67]. The tremendous increase in TFC (from 1.6 to 11.6 mg QE/g) and antioxidant activities in pea pods DES could be related to the liberation of soluble-bound dihydrochalcones [68]. Though DES also improved phenolic recovery in bean pods (29.9 to 47.6 mg GAE/g), this increase was less pronounced in pea pods. This suggests that bean pods already contain a higher proportion of free phenolics readily extractable with aqueous ethanol, and thus the contribution of bound conjugated forms is comparatively lower [69]. By contrast, artichoke bracts, known as an important source of caffeoylquinic acids, showed only a moderate enhancement after DES extraction. Further, Ozkan [70] reported that ChCl-Ox has a lower phenolic extraction yield compared to other hydrogen bond donors, such as lactic acid and sucrose. Limiting the incremental effect of DES can be explained by the fact that most chlorogenic acids are already present in soluble form, and extraction efficiency depends on other factors such as temperature and time [71].

**Table 3:** The antioxidant potential of vegetable waste powders, DES extract, and their corresponding bioplastic films as assessed by the *in vitro* tests of DPPH, ABTS, and FRAP tests.

Sample	TPC	TFC	DPPH	ABTS	FRAP
Powder extract					
Pea pods	7.1±0.2^d^	1.6±0.1^gh^	9.2±1.7^d^	11.0±0.5^e^	5.2±0.3^cd^
Bean pods	26.9±1.1^c^	3.3±0.2^d^	209.2±2.2^a^	62.9±1.1^a^	9.6±0.1^b^
Artichoke bracts	4.5±0.2^d^	2.1±0.1^fg^	13.5±0.5^d^	9.4±0.4^e^	5.9±0.3^cd^
DES extract					
Pea pods	60.0±9.4^a^	11.6±0.2^a^	90.7±6.6^c^	38.8±1.7^d^	10.7±0.7^b^
Bean pods	47.6±4.7^b^	9.4±0.2^b^	126.5±12.0^b^	46.5±1.4^b^	22.8±1.6^a^
Artichoke bracts	39.3±3.6^b^	4.1±0.1^c^	102.7±6.9^c^	42.8±1.7^c^	22.2±0.8^a^
Film extract					
Pea pods	3.3±1.9^d^	2.7±0.2^e^	15.7±3.2^d^	10.2±0.3^e^	6.6±0.3^c^
Bean pods	2.7±1.2^d^	2.3±0.1^ef^	11.7±1.5^d^	9.1±0.2^e^	5.7±0.2^c^
Artichoke bracts	3.7±0.2^d^	1.4±0.1^h^	9.2±0.9^d^	10.3±0.2^e^	4.4±0.2^d^

TPC and TFC are expressed as mg of gallic acid and quercetin equivalents/g of sample. DPPH, ABTS, and FRAP Activities are expressed as mg of Trolox equivalent / g of sample. Data are presented as mean±standard deviation (n = 3). Different superscript letters denote significant differences (p < 0.05) according to Tukey’s HSD test.

Interestingly, the bioplastic films retained a noticeable fraction of the DES-extracted phenolics from the raw materials. Pea pod–based films showed a modest enrichment in TFC (from 1.6 to 2.7 mg QE/g), which translated into significantly higher DPPH scavenging activity (15.7 mg TE/g) compared to the other films. In contrast, bean pod films displayed a sharp decline in phenolic content, reflecting a substantial loss of antioxidant capacity during incorporation into the newly formed polymer matrix. This decrease (−94 % and −85 % for DPPH and ABTS activities) can be attributed to the relative efficiency of ChCl-Oxalic acid in extracting the free soluble low molecular weight phenolics which are largely removed during DES recovery, leaving structurally bound phenolics,·associated·with·lignocellulosic·polymers.·This behavior is particularly pronounced in beans pods may be related to the predominance of a specific class of free phenolics (piscidic acid, vestitol, and hydroxyeucomic acid)[72], making them more susceptible to sequestration during DES processing and less soluble in the final assembly. Artichoke-based films exhibited only a slight DES treatment effect, consistent with the predominance of soluble phenolic acids that are more prone to diffusion into the new bioplastic matrix. Complementary experiments are needed to investigate these observations.

Although reduced compared to the corresponding DES extracts, the antioxidant activity of the films remains within or above the range reported for several DES-based antioxidant biopolymer films, such as ChCl/tannic acid soy films (DPPH TEAC ≈ 6.9 mg TE/g) and ChCl/citric acid alginate films incorporating chokeberry pomace or lemon balm (DPPH TEAC ≈ 0.8–2.9 mg TE/g)[73,74]. It is worth mentioning that retained activity is expected to be more relevant under contact-based or slow-release conditions, where matrix-bound phenolics can progressively interact with the surrounding medium, even if conventional solution-based *in vitro* assays underestimate their effective antioxidant performance [73,75].

### Antimicrobial activity

The antimicrobial properties of the three bioplastics were evaluated against *E. coli* ATCC 8739 (Gram-negative), *S. typhimurium* ATCC 14028 (Gram-negative), *S. aureus* ATCC 6538 (Gram-positive), *E. faecium* ATCC 19434 (Gram-positive), and *Candida albicans* ATCC 10231 (yeast), as shown in Fig. 5. The films (5 mm diameter) were tested by measuring the inhibition halo around the bioplastic discs.

**Fig. 5:**
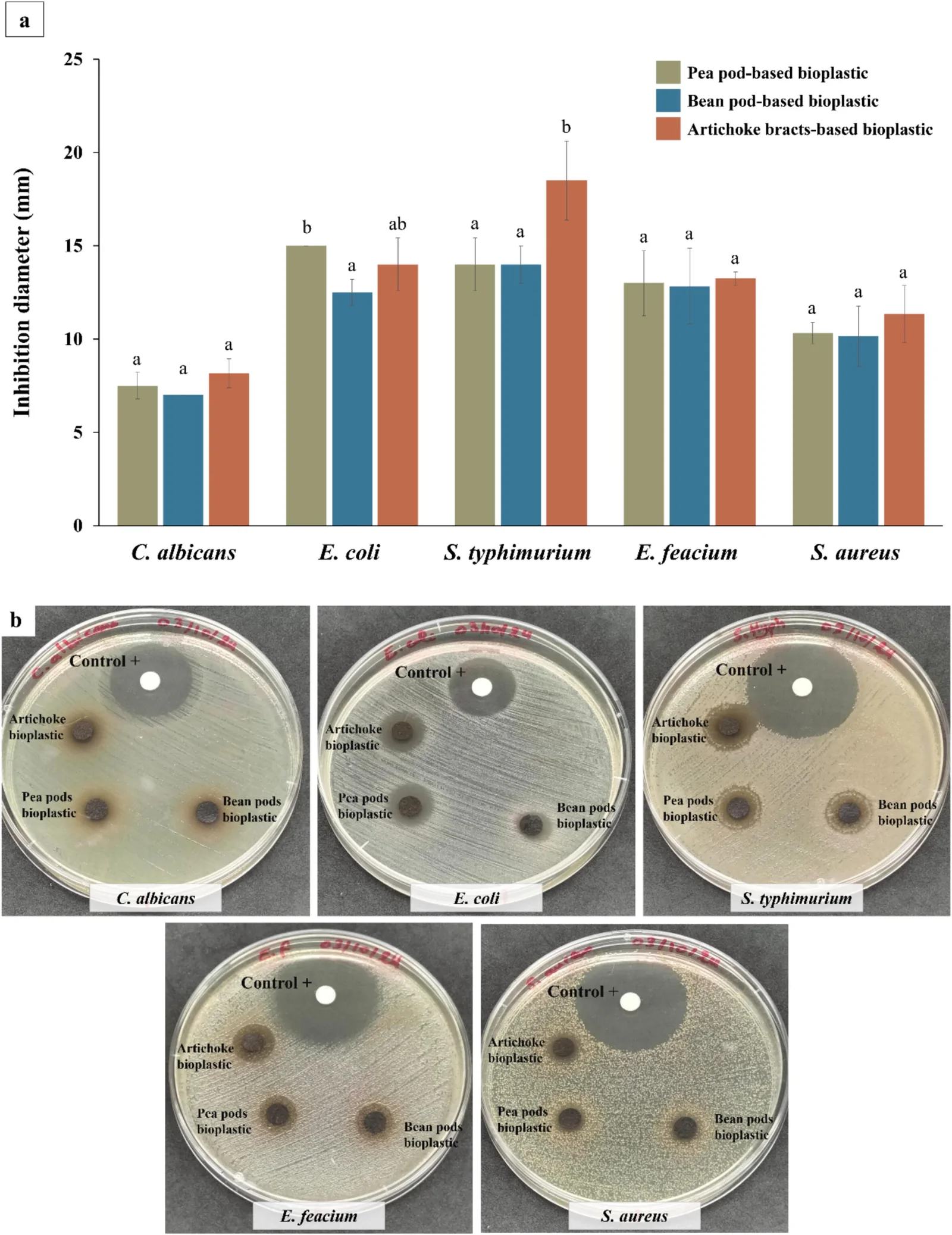
Antimicrobial activity of vegetable waste-based bioplastics against five pathogenic strains: (a) Inhibition zone diameters; (b) Representative Petri dish images showing inhibition halos.

All tested films exhibited measurable antimicrobial activity, with inhibition zones particularly pronounced against *S. typhimurium* and *E. faecium*. This bioactivity can be attributed to naturally occurring phytochemicals (e.g., polyphenols, flavonoids, organic acids, and fibers) inherently present in vegetable by-products [76], which are retained within the biopolymer matrix and can diffuse into the surrounding medium to inhibit microbial growth. The artichoke bract-based bioplastic showed the strongest antimicrobial effect, whereas activity against *C. albicans* was consistently the lowest (Fig. 5a). This variability can be explained by differences in microbial cell wall structures. Gram-negative *S. typhimurium*, despite its outer membrane, appears highly sensitive to certain active compounds released from the films, while *C. albicans*, with its chitin- and glucan-rich cell wall, provides stronger resistance [13].

When compared with other bioplastic films reported in the literature, the vegetable processing waste films developed in this study exhibit markedly stronger antimicrobial performance. For instance, corn-starch composites with essential oils or nanoparticles typically demonstrated inhibition zones of 6–14 mm against *E. coli* and *S. aureus* [77]. Banana-starch bioplastic films similarly produce small halos, often below 10 mm in diameter [78]. In contrast, artichoke bract-based bioplastic produced significantly larger inhibition halos against both Gram-positive and Gram-negative strains (Fig. 5b), indicative of superior antimicrobial efficacy.

### Biodegradability

To evaluate the environmental performance of the synthesized lignocellulosic bioplastics, they were subjected to a soil burial test and compared with conventional PVC films as a non-biodegradable control. All samples were buried in moist soil under controlled laboratory conditions, and their physical and morphological changes were monitored for 70 days (Fig. 6).

**Fig. 6:**
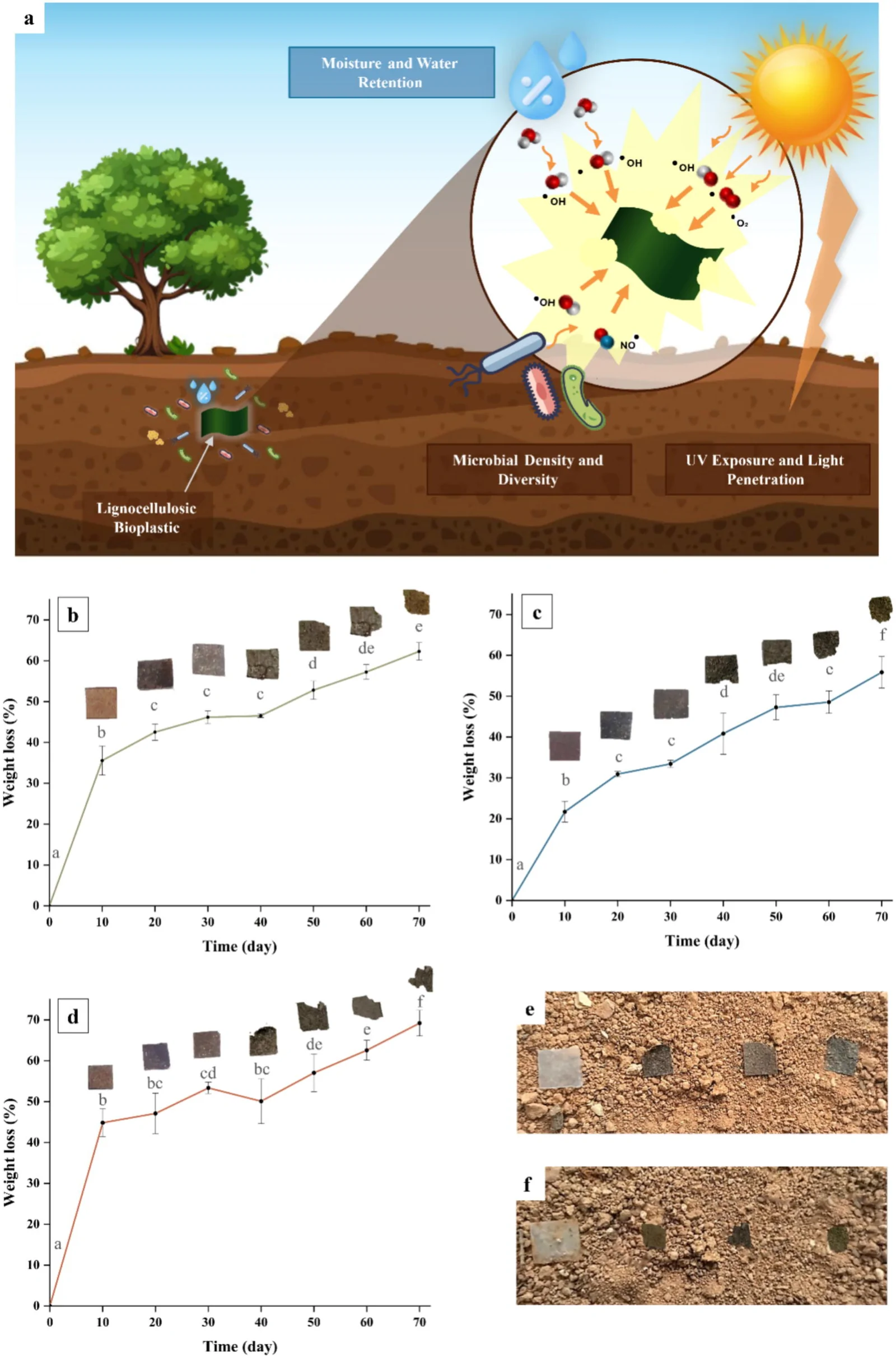
Soil biodegradability of lignocellulosic bioplastics. (a) Schematic illustration of the biodegradation mechanism. (b–d) Weight loss profiles of pea pod-, bean pod-, and artichoke bract–based bioplastics during 70 days of soil burial. (e, f) Visual appearance of the bioplastics before and after soil burial, respectively.

During the first 20–30 days, the lignocellulosic bioplastics exhibited superficial changes such as gradual surface discoloration and roughening. These modifications are typically associated with early microbial activity; however, the films largely retained their overall structural integrity during this period. This initial resistance can be attributed to the intrinsic antimicrobial properties of the bioplastics, which likely slowed down deep microbial colonization and delayed extensive deterioration. Similar effects have been observed in other bio-based films containing natural phenolics [79]. By day 70, the films showed substantial degradation characterized by edge weakening, surface erosion, and partial fragmentation (Fig. 6f), aligning with degradation behavior reported for other lignocellulosic materials under composting or native soil conditions [80]. In contrast, PVC samples preserved their original structure and color throughout the test, confirming their non-biodegradable nature. Quantitative weight-loss measurements further supported these observations. Indeed, the bioplastics derived from pea pods, bean pods, and artichoke bracts exhibited 62.3 %, 55.9 %, and 69.2 % mass loss, respectively, after 70 days, whereas PVC films showed no measurable change. These results highlight a desirable balance in the performance of the lignocellulosic bioplastics, providing sufficient structural stability during the early stages of use while ensuring efficient biodegradation upon disposal.

## Conclusion

This study demonstrates that the native lignocellulosic composition of vegetable processing residues directly governs the structural, mechanical, barrier, and functional properties of the resulting bioplastics, and that DES-mediated in situ lignin regeneration is an effective tool for tailoring these properties. Using artichoke bracts, pea pods, and bean pods for the first time as feedstocks in this approach, choline chloride-oxalic acid DES enabled controlled cellulose reorganization, hemicellulose depolymerization, and lignin regeneration without fractionation. Molecular docking provided novel molecular-level insight into lignin-cellulose interactions, revealing oxalic acid-mediated hydrogen bonding as a key contributor to matrix cohesion and performance. The resulting bioplastics combined UV–Vis light shielding, bioactivity, and rapid biodegradability (56–69 % within 70 days), with composition-dependent performance suited to flexible and semi-rigid packaging applications. Future work will focus on process upscaling, long-term stability, and functional optimization to further advance these materials toward industrial implementation within a circular bioeconomy framework.

## Compliance with ethics requirements

This article does not contain any studies with human or animal subjects.

## CRediT authorship contribution statement

**Cyrine Amara:** Methodology, Investigation, Visualization, Data curation, Formal analysis, Writing – original draft. **Safa Baraketi:** Methodology, Investigation, Visualization, Data curation, Formal analysis, Writing – original draft. **Yosr Barchouchi:** Methodology, Investigation, Visualization, Data curation, Formal analysis. **Yassine M’Rabet:** Methodology, Investigation, Writing – review & editing, Supervision. **Wiem Ayadi:** Methodology, Investigation, Visualization. **Mabrouk Horchani:** Methodology, Investigation, Writing – review & editing. **Khaoula Khwaldia:** Methodology, Investigation, Visualization, Data curation, Formal analysis, Writing – review & editing, Supervision, Funding acquisition, Resources.

## Declaration of competing interest

The authors declare that they have no known competing financial interests or personal relationships that could have appeared to influence the work reported in this paper.
